# The Utility of Scoring Systems in Predicting Early and Late Mortality in Alcoholic Hepatitis: Whose Score Is It Anyway?

**DOI:** 10.1155/2012/624675

**Published:** 2012-09-04

**Authors:** Naaventhan Palaniyappan, Venkataraman Subramanian, Vidyasagar Ramappa, Stephen D. Ryder, Philip Kaye, Guruprasad P. Aithal

**Affiliations:** NIHR Biomedical Research Unit in Gastrointestinal and Liver Diseases, Nottingham University Hospitals NHS Trust and The University of Nottingham, Nottingham NG7 2UH, UK

## Abstract

*Background*. Alcoholic hepatitis (AH) is a distinct clinical entity in the spectrum of alcoholic liver disease with a high short-term mortality. Several scoring systems are being used to assess the severity of AH but the ability of these scores to predict long-term survival in these patients is largely unknown. *Aims*. We aim to assess the utility of five different scoring systems Child Pugh (CP), model for end-stage liver disease (MELD), Maddrey's discriminant function (mDF), Glasgow AH score (GAHS), and age-bilirubin-INR-creatinine (ABIC) score in predicting shot-term and long-term survival in patients with AH. *Methods*. Patients with histological evidence of AH were identified from our database. The clinical and biochemical parameters were used to calculate the 5 different scores. The prognostic utility of these scores was determined by generating an ROC curve for survival at 30 days, 90 days, 6 months, and 1 year.* Results and Conclusions*. All 5 scores with the exception of CP score have a similar accuracy in predicting the short-term prognosis. However, they are uniformly poor in predicting longer-term survival with AUROC not exceeding 0.74. CP score is a very poor predictor of survival in both short and long term. Abstinence from alcohol was significantly (*P* < 0.05) associated with survival at 1 year.

## 1. Introduction

Alcoholic hepatitis (AH) is one of the most recognised “acute on chronic” liver syndromes, wherein patient presents with symptoms and signs of acute decompensation with evidence of chronic liver disease, in the setting of ongoing or recent consumption of excess alcohol [1]. Patients present with progressive jaundice, tender hepatomegaly, and evidence of systemic inflammatory response (SIRS) with characteristic liver biopsy findings of ballooned hepatocytes and Mallory bodies (eosinophilic inclusion bodies) surrounded by neutrophils [2]. AH is a cause of considerable mortality and morbidity in the Western population. A Danish study reported a 28-day mortality rate of 15% among patients hospitalised for AH [3]. A pooled one-month mortality of patients with AH who were treated with placebo in randomised control trials (RCTs) was 22.44% in US and 18.45% in Europe [4]. Short-term prognosis of alcoholic hepatitis is worse than that of decompensated cirrhosis as defined by the system agreed at the Baveno IV consensus conference; 1-year probability of mortality is 20% in decompensated cirrhosis [5]. Hence, it is important to distinguish patients with AH, in particular those with much worse short-term prognosis, from those with decompensated cirrhosis so that the former group are targeted for specific potentially effective treatments [6–8].

Several scoring systems have been developed and used to assess the severity of AH and to predict survival in these patients. Maddrey's discriminant function (DF) has been used in clinical practice for more than 30 years [9]. A DF of 32 is used to stratify a patient's severity of AH, patients with a score of ≥32 having a high short-term mortality [10]. Model for end-stage liver disease (MELD) score was initially developed to predict survival in patients with cirrhosis and portal hypertension, but was found to detect short-term survival in patients with AH with good accuracy [11]. However, the cut-off value for MELD score in detecting severe AH is still controversial with various studies using different values to assess the accuracy of the score. The Glasgow alcoholic hepatitis (GAH) score identifies the subgroup of patients with a DF of >32 who will recover without steroids [12]. The ABIC score was developed to categorise patients with AH into high-, moderate-, and low-risk groups based on the risk of death at 90 days and 1 year [13]. The Lille score evaluates the response in serum bilirubin after a 7-day course of corticosteroid therapy and aids the decision in either stopping the corticosteroids or completing a 28-day course [14]. The prognostic value of portal pressure, measured by hepatic venous pressure gradient (HVPG), in chronic liver disease has been well recognised [15, 16]. However, the influence of portal pressure in AH as a distinct entity itself has not been established.

 The ability of these various scores to predict long-term survival in patients with AH is largely unknown. Therefore, we attempt to assess the utility of the different published scoring systems in predicting short-term (30 and 90 days) and long-term (6 and 12 months) survival in patients with AH.

## 2. Methods

We identified patients with the diagnosis of AH from our histological database. The decision to refer patients for liver biopsy was made by the primary physician, which was a hepatologist in all cases. As it is our practice to perform liver biopsy through transjugular approach whenever alcoholic hepatitis is suspected, the histological findings, all the transjugular liver biopsies performed in Queens Medical Centre (QMC), Nottingham University Hospitals between 2004 and 2007, were reviewed from the online hospital database. Biopsies showing histological evidence of AH (Table 1) as determined by a single pathologist (PK) were included in the analysis. All the patients undergoing transjugular biopsies have their HVPG measured simultaneously and this was used to identify the degree of portal hypertension in these patients.

The clinical and biochemical parameters for these patients with biopsy-proven AH was collected from the patient notes and electronic database. These parameters were used to calculate the scoring systems that have been described to guide the management of patients with AH. The measurement units of the biochemical parameters were converted to the relevant units as dictated by the derivation formula of these scores.

Abstinence from alcohol in the short term (3–6 months from the histological diagnosis of AH) among these patients was determined during their follow-up clinic appointment or any hospital admissions in this period.

Short-term (30 and 60 days) and long-term (90 days and 1 year) survival was evaluated in these patients. The prognostic value of the scoring systems was determined by generating a receiver operating (ROC) curve and the area under the curve was calculated.

## 3. Results

Over the study period, 140 transjugular liver biopsies were performed in QMC. 44 of these biopsies showed histological evidence of AH. The patient demographics and characteristics of their biopsies are summarised in Table 2. Almost all the biopsies demonstrated steatosis and neutrophil infiltration with over half of them having Mallory bodies and swollen hepatocytes. 40.9% (18/44) of the biopsies showed evidence of underlying cirrhosis.

The clinical and biochemical parameters of these 44 patients with biopsy-proven AH were used to calculate the various scoring systems that have been proposed (Table 3). The HVPG measurement was not available for a single patient due to malfunctioning instruments during the transjugular liver biopsy.

Follow-up data for one patient was not available as the patient's care was transferred to another hospital. The cumulative 30-day and 90-day mortality in this subgroup was 11.6% (5/43) and 14.0% (6/43). The long-term survival data was unavailable for another patient whose followup was lost. The respective 6-month and 1-year cumulative mortality in this cohort was 21.4% (9/42) and 26.2% (11/42).

In predicting the short-term (30 and 90 day) prognosis in this cohort of patients, GAHS, Maddrey's DF, MELD, and ABIC scores all have a similar accuracy as demonstrated by their AUROC. However, they are uniformly poor in predicting survival beyond 6 months with AUROC not exceeding 0.74. Childs-Pugh score has been shown to be a very poor predictor of survival in both short and long term (Table 4). Clinically significant portal hypertension (HVPG ≥ 10 mmgHg) is neither associated with short-term nor long-term prognosis (*P* = nonsignificant).

Abstinence from alcohol in 3 to 6 months from the diagnosis of AH was significantly associated with survival at the end of the year (*P* < 0.05) and predicted survival with an AUROC of 0.83 (95 CI: 0.71–0.95).

## 4. Discussion and Conclusion

Despite decades of debate and controversy, the role of histology in identifying the specific cohort described in these studies is still not unequivocally established. In a study that analysed 41 patients biopsied within a month of first presentation with decompensated alcoholic liver disease, none of the histological features were predictive of survival by Cox multivariate analysis [17]. In contrast, a recent study showed that the positive likelihood ratio of the presence of SIRS and clinical features in diagnosing AH is only 1.2, while histological criteria had the best area under the curve in the prediction of adverse outcome [18]. In addition to the lack of consensus, patients with AH have low platelet count and coagulopathy necessitating transjugular approach for the liver biopsy. As transjugular liver biopsy is available in limited number of centres, algorithms and scores using clinical and simple laboratory parameters are widely used in the clinical management of these patients.

We have described a comparison of 5 different prognostic scores in their value of predicting short- and long-term survival in patients with AH. All the scores with the exception of the CP score have a reasonable accuracy in predicting 30- and 90-day mortality. CP score was originally described to assess the operative risk is patients with established cirrhosis and was developed to predict their survival [19]. The relative ease by which it can be calculated at the bedside has meant that it has remained popular among clinicians. However, its use is limited in predicting prognosis in patients with ‘‘acute on chronic liver failure,” in particular those with alcoholic hepatitis.

As with CP score, MELD is not a system developed specifically to evaluate AH. MELD score was described initially to predict survival following elective transjugular intrahepatic portosystemic shunts (TIPSS) for the prevention of variceal rebleeding or for the treatment of refractory ascites [20]. Utility of MELD score has since extended to assess the mortality risk in patients with end-stage liver disease and to aid organ allocation priorities in transplant centres [11]. Strength of MELD score is that it functions as a continuous variable and hence the clinical outcome can be accurately estimated based on a particular individual's MELD score. In our cohort, MELD performs well in predicting short-term outcome in AH. However, estimation of MELD score requires the use of a calculator and there is no consensus on the optimal cut-off value for this score as different studies have chosen different tradeoffs in setting the test threshold (sensitivity and specificity) [21–24].

Value of Maddrey's DF has been verified by more than 30 years experience [10]. Its main use has been in determining the group of patients with AH that might benefit from corticosteroid therapy. However, Maddrey's DF has often been used to assess the severity of biopsy proven AH and this has not yet been accepted as a standard practice. The GAH score depends entirely on simple clinical and laboratory parameters and has been shown to have a higher overall accuracy compared to the MELD and Maddrey's DF in predicting in-hospital death [12]. It also stratifies the group of patients with a high Maddrey's DF who will recover without being treated with steroids. The ABIC score was developed in an attempt to risk stratify the death in patients with AH at 90 days and 1 year [13]. It stratifies patients into high-, moderate-, and low-risk groups. In our study, the performance characteristics of both GAH and ABIC scores were comparable to that of Maddrey's DF. However, the GAH and ABIC scores have not been verified in countries out of which they were derived in.

The influence of portal pressure in the setting of alcoholic hepatitis is not well established. Rincon et al. attempted to evaluate the prognostic value of HVPG in patients with acute alcoholic hepatitis and Maddrey's DF of greater than 32 [25]. HVPG of more than 22 mmHg was found to be an independent predictor of in-hospital mortality. Long-term survival was not evaluated in this cohort. In our study, we failed to demonstrate any association between clinically significant portal hypertension (HVPG ≥ 10 mmHg) with short- and long-term survival.

Our study has demonstrated that majority of the prognostic scores are comparable in their performance characteristics for predicting short-term mortality in patients with AH, and hence, are of similar utility in clinical practice. However, all the scores that we evaluated are uniformly poor in predicting longer-term survival beyond 6 months. We have shown that the abstinence in the first 3 months following the diagnosis of AH is associated with survival at the end of 1 year. It has been established that abstinence from alcohol is the most important intervention for patients with alcoholic liver disease (ALD). Abstinence has been shown to improve outcome and histological features in all stages of ALD, with improvement noticed in 3 months [26]. As alcohol abstinence is important in improving long-term survival, clinicians should remain focussed in ensuring that these patients have the opportunity to be abstinent from alcohol and continue to do so. The role of pharmacological agents in helping to sustain abstinence is still unclear and requires further investigation.

## Figures and Tables

**Table 1:** Histological features in alcoholic hepatitis.

(i) Steatosis	
(ii) Ballooned hepatocytes	
(iii) Lobular inflammation	
(iv) Eosinophilic inclusion bodies—Mallory bodies	
(v) Neutrophil infiltration	
(vi) Megamitochondria	

**Table 2:** Patient demographics and histological findings in the 44 biopsy proven alcoholic hepatitis.

Sex	
Male	25 (56.8%)
Female	19 (43.2%)
Mean age, in years	48
Liver biopsy findings	
Steatosis	42 (95.4%)
Necrosis	10 (22.7%)
Neutrophil infiltration	43 (97.7%)
Mallory bodies	26 (59.1%)
Ballooned hepatocytes	29 (65.9%)
Acidophilic bodies	2 (4.5%)
Giant mitochondria	1 (2.27%)
Underlying stage of liver disease	
Cirrhosis	18 (40.9%)

**Table 3:** Prognostic scores of 44 patients with histological evidence of AH at the time of biopsy.

Prognostic scores	*N*/Median
Child-Pugh score (±SD)	10.5 (±2.38)
A (%)	5 (11.4%)
B (%)	8 (18.2%)
C (%)	31 (70.6%)
Model of end-stage liver disease (MELD) (±SD)	18.5 (±6.51)
Maddrey's discriminant factor (MDF) (±SD)	31.6 (±19.7)
Glasgow alcoholic hepatitis (GAH) score (±SD)	7 (±1.47)
ABIC score	7.19 (±1.47)
HVPG, mmHg (±SD)	13 (±6.47)

**Table 4:** The area under the receiver operating characteristic (AUROC) for prognostic scores for short- and long-term mortality in patients with AH.

Prognostic score	30-day mortality		90-day mortality		6-month mortality		1-year mortality	
	AUROC	95% CI	AUROC	95% CI	AUROC	95% CI	AUROC	95% CI
CP	0.53	0.25–0.8	0.47	0.21–0.73	0.55	0.35–0.76	0.5	0.31–0.69
mDF	0.79	0.64–0.94	0.81	0.67–0.95	0.72	0.54–0.91	0.63	0.43–0.82
GAHS	0.78	0.54–1	0.81	0.61–1	0.73	0.54–0.92	0.64	0.44–0.84
ABIC score	0.74	0.46–1	0.79	0.55–1	0.67	0.44–0.91	0.66	0.45–0.87
MELD	0.84	0.71–0.96	0.85	0.74–0.97	0.74	0.56–0.92	0.64	0.44–0.83
